# Emergence and genomic insights of non-pandemic O1 *Vibrio cholerae* in Zhejiang, China

**DOI:** 10.1128/spectrum.02615-23

**Published:** 2023-10-11

**Authors:** Yun Luo, Michael Payne, Sandeep Kaur, Sophie Octavia, Jianmin Jiang, Ruiting Lan

**Affiliations:** 1 School of Biotechnology and Biomolecular Sciences, University of New South Wales, Sydney, New South Wales, Australia; 2 Zhejiang Provincial Center for Disease Control and Prevention, Hangzhou, Zhejiang, China; 3 Key Lab of Vaccine, Prevention and Control of Infectious Disease of Zhejiang Province, Hangzhou, Zhejiang, China; University of Maryland Eastern Shore, Princess Anne, Maryland, USA

**Keywords:** non-pandemic O1, *Vibrio cholerae*, genomic epidemiology, T3SS

## Abstract

*Vibrio cholerae* O1 has caused cholera pandemics. Non-pandemic *V. cholerae* O1 strains, which are genetically distinctive from the pandemic clones, have been isolated from both human infections and the environment. We aimed to better understand the non-pandemic O1 strains and their pandemic potential. We sequenced 109 non-pandemic O1 isolates from Zhejiang, China (from 1963 to 1996) and compared them with 62 publicly available non-pandemic O1 genomes. The isolates from Zhejiang can be classified into three lineages (L1–L3). All grouped together with L3 sharing the most recent common ancestor with the pandemic clones. L2 and L3 emerged in the 1960s while L1 emerged in the 1970s. L1 and L2 disappeared after the 1990s, but L3 persisted until recently. All isolates contained the type VI secretion system. The *Vibrio* pathogenicity island was present in all L3 isolates, whereas the type III secretion system was present in all L1 isolates. L2 did not carry any unique virulence genes. An intact CTXφ was present in only two L3 isolates. An intact *Vibrio* seventh pandemic island 1 was present in only three L3 isolates. The *bla_CARB-7_* gene was identified in 96.3% of L2 isolates. Each of the non-pandemic O1 lineages has unique properties contributing to their capacity to cause disease. Our findings offer new insight into the evolution of O1 *V. cholerae* for cholera prevention and control.

**IMPORTANCE:**

It is well recognized that only *Vibrio cholerae* O1 causes cholera pandemics. However, not all O1 strains cause pandemic-level disease. In this study, we analyzed non-pandemic O1 *V. cholerae* isolates from the 1960s to the 1990s from China and found that they fell into three lineages, one of which shared the most recent common ancestor with pandemic O1 strains. Each of these non-pandemic O1 lineages has unique properties that contribute to their capacity to cause cholera. The findings of this study enhanced our understanding of the emergence and evolution of both pandemic and non-pandemic O1 *V. cholerae*.

## INTRODUCTION

Cholera is an acute diarrheal disease that can be fatal without proper treatment (1). Since 1817, there have been a series of seven recorded cholera pandemics. The current seventh cholera pandemic began in Indonesia in 1961 and spread through Asia and then to other continents (2). The etiological agent of cholera is *Vibrio cholerae*, which is a Gram-negative bacterium and is comprised of more than 200 serogroups. Only O1 and O139 serogroups have been reported to cause pandemic or epidemic cholera (3). All serogroups other than O1 and O139 are collectively referred to as non-O1/non-O139 *V. cholerae,* which have been found in the environment and sporadically cause human infections (4, 5).

The main virulence factor of *V. cholerae* causing diarrhea is cholera toxin (CT), which is encoded by the *ctxAB* genes located on the CTX phage (CTXφ). The *Vibrio* pathogenicity island (VPI) is another major virulence element in *V. cholerae*. The VPI contains 29 genes including genes encoding the toxin coregulated pilus (TCP) and the accessory colonization factor (ACF) (6). The major protein and highly variable subunit of TCP is TcpA (7). Additionally, a type VI secretion system (T6SS) and a type III secretion system (T3SS) are widely found in *V. cholerae* (8 – 10). The T3SS gene cluster is more sporadically present in non-O1/non-O139 isolates (4), and T3SS is found to play a key role in diarrheal disease caused by non-O1/non-O139 *V. cholerae* in an infant rabbit model (11).

*V. cholerae* is a highly diverse species. There are 1,505 sequence types (STs) by multilocus sequence typing (MLST) in the PubMLST database (8 February 2022) (12). The seventh pandemic clone consists of ST69 and ST515 (13, 14), while the sixth pandemic clone, pre-seventh pandemic clone, Australian clone, and the U.S. Gulf Coast clone belong to ST73, ST71, ST70, and ST75, respectively (12). Cholera cases caused by O1 serogroup ST75 have also been reported in China (15, 16) and Africa (17). The sub-lineages of ST75 are geographically widespread with most carrying both the VPI and the CTXφ (17). Fourteen other O1 STs including ST169 and ST170 have been reported previously (15). In this study, we refer to O1 STs unrelated to the pandemic clones as non-pandemic O1 STs.

However, little is known of these non-pandemic O1 STs in terms of their genomic diversity, their relationship to pandemic clones, and their mechanisms of causing disease. Not all O1 clones possess CTXφ to cause disease (18). Non-toxigenic O1 (*ctxAB* negative) isolates were observed in both the environment and patients in other studies (18, 19). It has been suggested that non-toxigenic O1 in the environment can gain virulence genes from the epidemic clone to become epidemic strains (20). However, the relationship between non-pandemic or non-toxigenic *V. cholerae* O1 isolates and the pandemic lineages remained largely unclear.

In this study, we sequenced 109 *V*. *cholerae* non-pandemic O1 isolates from Zhejiang, China, isolated from the 1960s, the early stage of the seventh pandemic, to the 1990s. We investigated the phylogenetic relationship between non-pandemic isolates and the seventh pandemic clone and their genomic content in relation to virulence. This study aimed to better understand the diversity and pathogenic mechanisms of non-pandemic *V. cholerae* O1 and shed light on the genomic evolution of all *V. cholerae* serogroup O1 clones.

## MATERIALS AND METHODS

### Bacterial isolates

A total of 109 isolates obtained from 1963 to 1996, which were archived in Zhejiang Provincial Centre for Disease Control and Prevention, were cultured on nutrient agar. All isolates were confirmed as *V. cholerae* O1 serogroup using API 20NE (bioMérieux, France) and *V. cholerae* polyvalent antiserum, serovar Inaba, or Ogawa antiserum (Denka Seiken, Japan).

### Whole-genome sequencing (WGS)

DNA of all 109 isolates were extracted from overnight cultures using a QIAamp DNA mini kit (Qiagen, German). The 150-base paired-end sequencing library was constructed using TruePrep DNA Library Prep Kit V2 (Illumina, Santiago, CA, USA). WGS was performed using the Illumina Hiseq X Ten sequencing platform.

### Genome selection

We downloaded all public *V. cholerae* raw-read sequences from the European Bioinformatics Institute (EBI) European Nucleotide Archive (ENA) database (24 May 2021). SPAdes (21) was used to assemble the genomes. Two criteria were used to identify non-pandemic O1 isolates. Firstly, Nucleotide BLAST was used to screen for all the genomes that were O1 *rfbV* gene positive and O139 *wbfR* gene negative (22). Secondly, MLST was used to identify isolates that did not belong to a pandemic and closely related strains including ST69/ST515 (seventh pandemic STs), ST73 (sixth pandemic ST), ST71 (pre-seventh pandemic ST), ST75 (U.S. Gulf Coast clone ST) and ST70 (Australian clone ST) (12). A total of 702 genomes from ENA and 109 genomes from Zhejiang passed the criteria and were used in this study as non-pandemic O1 isolates. We also included seven genomes that were O1 *rfbV* gene positive but O139 *wbfR* gene negative and typed as ST69/ST515 (seventh pandemic STs), ST73 (sixth pandemic ST), ST71 (pre-seventh pandemic ST), ST75 (U.S. Gulf Coast clone ST), and ST70 (Australian clone ST) to represent the pandemic and closely related lineages.

### Phylogenetic analysis and pan-genome analysis

We used the pipeline SaRTree (23) to do SNP calling and removed all recombinant SNPs. The SNPs of the genomes were called by aligning and mapping separately against the large and small chromosomes of the reference genome, which was a *V. cholerae* O1 biovar El Tor strain N16961 (GenBank accession no. GCF_900205735.1). IQ-Tree (version 2.0.4) (24) with default parameters (best-fit model: TVM + F + ASC + R4) and 1,000 ultrafast bootstraps (25) was used to construct the maximum likelihood tree using the SNPs from both chromosomes. The *Vibrio paracholerae* strain BH2680 was used to root the phylogeny. The Newick files obtained from IQ-Tree were annotated and visualized in iTOL (version 6.5.2) (26). SNPs were annotated on each branch of the maximum likelihood tree by the SaRTree pipeline.

The pan-genome calculation was performed by Roary v3.11.2 (27) with thresholds of 96% for protein BLAST (BLASTP) percentage identity and pan genes data were visualized in R script. Scoary v1.6.16 (27) was used to calculate the accessory genes associated with different lineages.

### Multilevel genome typing (MGT)

All raw reads of the 109 isolates in this study were processed and submitted to the *V. cholerae* MGT database according to its documentation (28). MGT STs were assigned and visualized automatically by the MGTdb site (https://mgtdb.unsw.edu.au/vibrio/).

### Genetic element analysis

All analyses were performed on 181 genomes that had associated metadata in this study. The ABRicate pipeline (Seemann T, Abricate, Github https://github.com/tseemann/abricate) with databases of NCBI ResFinder (29) and PlasmidFinder (30) was used to predict the antimicrobial resistance (AMR) genes and plasmids, respectively, from the genomes. VFDB database (31) in ABRicate and a customized database were used to predict virulence genes. KMA (32) was also used to identify these virulence and housekeeping genes from raw reads. Housekeeping gene coverage was used as a control for the average depths of virulence genes. Two criteria were used for gene presence. One was the minimum identity and coverage threshold of 80% from ABRicate. The other one was the ratio of virulence gene depth to the average depth of housekeeping genes greater than 20% from KMA (10). To compare the virulence-related islands in *V. cholerae* (VPI, VSP-1, and VSP-2) among different clones, we used BLAST against the sequences of reference strain N16961 and extracted the intact island sequences from genomes to do the alignment.

## RESULTS

### Serotyping and *in silico* MLST typing of Zhejiang non-pandemic O1 isolates

**Fig 1:**
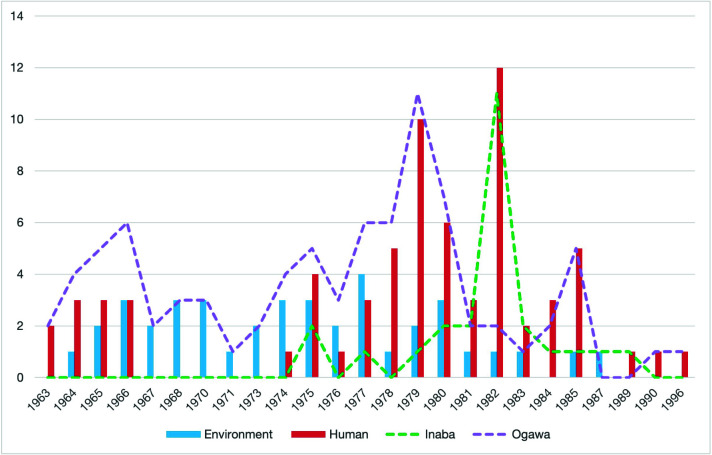
Distribution of source, year, and serotype of the Zhejiang *V. cholerae* O1 isolates used in this study. The *y*-axis shows the number of isolates, while the *x*-axis shows the year of isolation. The color bars are the isolate numbers from different sources in each year as shown in the legend. The dash lines per colour legend show the number of O1 isolates by serotype (Inaba or Ogawa) in each year.

A total of 109 isolates from human and environmental sources (Table S1) from 1963 to 1996 in Zhejiang, China, were sequenced in this study. The number of isolates from 1975 to 1982 was higher than that during other years, and the isolates were mainly obtained from humans. By serotyping, 77.1% of the isolates were identified as serotype Ogawa, and 22.9% were identified as serotype Inaba (Fig. 1). A large proportion (44%) of the serotype Inaba isolates were isolated in 1982, while the others were from 1975 to 1989.

**Fig 2:**
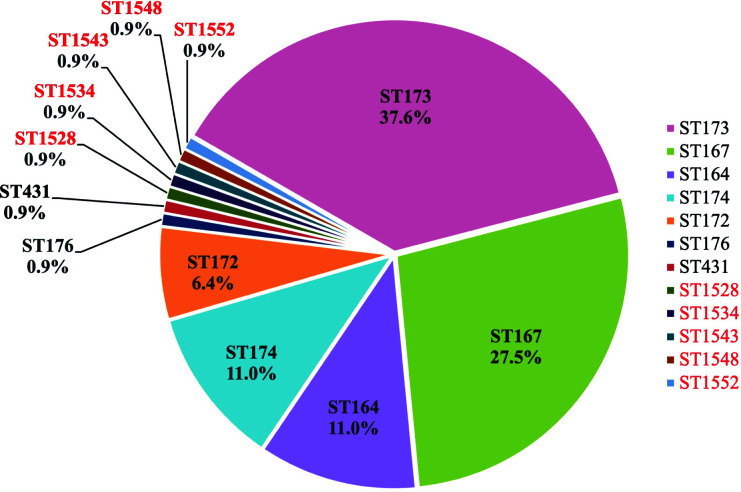
Sequence types (STs) of Zhejiang isolates. STs and the percentages in this study are shown in different colors. The new STs were in red.

*In silico* MLST typed the isolates into seven known STs (ST164, ST167, ST172, ST173, ST174, ST176, and ST431) and five new STs (ST1528, ST1534, ST1543, ST1548, and ST1552). ST167 and ST173 were the major STs accounting for 65.1% of the isolates (Fig. 2).

### Phylogenetic analysis

**Fig 3:**
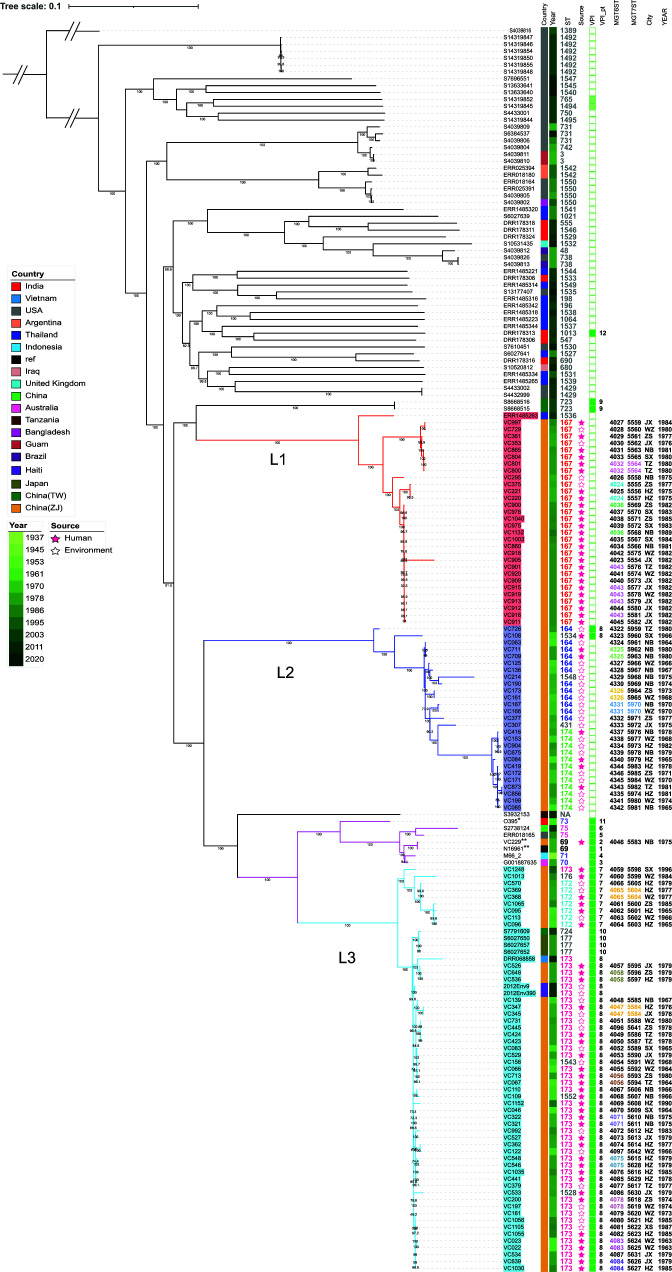
Phylogenetic tree of three lineages from Zhejiang and other publicly available clones of *V. cholerae* O1.The tree was rooted using *V. paracholerae* strain BH2680 as an outgroup. Lineage 1 (**L1**), Lineage 2 (**L2**), and Lineage 3 (**L3**) were demarcated with red, purple, and cyan branches, respectively. The isolates with orange labels were from Zhejiang. * represents the strain of the sixth pandemic (classic biotype), and ** represents the strains of the seventh pandemic (El Tor biotype). On the right side of the tree were metadata and genetic information as shown on the heading of each column. Filled and empty green squares represent the presence and absence of VPI genes, respectively. Light green squares represent the presence of partial genes on the VPI.

We screened publicly available *V. cholerae* genomes for O1 isolates based on the presence of the O1 *rfbV* gene and absence of the O139 *wbfR* gene and identified 702 genomes. We further typed these genomes by *in silico* MLST to remove pandemic and closely related STs (seventh pandemic ST69 and ST515, sixth pandemic ST73, pre-seventh pandemic ST71, U.S. Gulf Coast clone ST75, and Australian clone ST70). We further excluded genomes without metadata (year and/or location of isolation), 65 of the 702 publicly available O1 genomes were identified as non-pandemic O1 genomes and included in phylogenetic analysis (Fig. 3). In addition, five genomes representing the pandemic and closely related clones (ST69, ST70, ST71, ST73, ST75) were also included in the phylogenetic analysis. Zhejiang isolates fell into three lineages (L1–L3) (Fig. 3). Note that the lineage numbering in this study is independent of lineage numbering in Mutreja et al. (33), who assigned lineage names to pandemic and closely related strains. Of the 65 publicly available genomes, eight fell into the three lineages (L1–L3) with one in L1 and seven in L3, while the remaining 56 were located outside the three lineages belonging to 41 different STs. L3 was grouped together with the lineage containing pandemic and closely related clones. We also included 145 non-O1 isolates representative of 145 STs to construct a phylogenetic tree and found that the three lineages were grouped together without interweaving non-O1 isolates (Fig. S1), suggesting the three lineages shared a most recent common ancestor (MRCA), which acquired the O1 antigen gene cluster as one event.

L1 consisted of 30 ST167 isolates from Zhejiang and one ST1536 isolate from Thailand. ST167 was the dominant ST in L1 (30/31). Four isolates in L1 were environmental, and 26 were clinical. The source of the Thailand isolate was not known. L2 had a total of 27 isolates, composed of 12 ST164 isolates, 12 ST174 isolates, and one isolate each for ST431, ST1534, and ST1538. All L2 isolates were from Zhejiang. Most isolates in L2 were obtained from the environment (20/27) with seven from humans (7/27). L3 was the largest lineage with 52 isolates from Zhejiang, three from Japan, two from Haiti, one from Taiwan, and one from Vietnam. ST173 was the dominant ST (44/59), followed by ST172 (7/59). ST176, ST177, and ST724 were also grouped in this lineage. Three isolates from Zhejiang in L3 belonged to new STs (ST1528, ST1543, and ST1552). In L3, 16 Zhejiang isolates were from the environment, while 36 Zhejiang isolates were from humans. The sources of the non-Zhejiang isolates were unknown except for two Haitian isolates, which were from the environment.

We assigned SNPs to the branches of the phylogenetic tree (Fig. S2) and found that the lineage divisions were well supported by SNPs. L1 was supported by 278 SNPs on branch 229, and L2 was supported by 204 SNPs on branch 293. L3 was supported by 163 SNPs on branch 5, while the pandemic clones were supported by 58 SNPs on branch 4. L3 shared a common ancestor with both the seventh pandemic clone and the sixth pandemic clone supported by 40 SNPs on branch 118.

**Fig 4:**
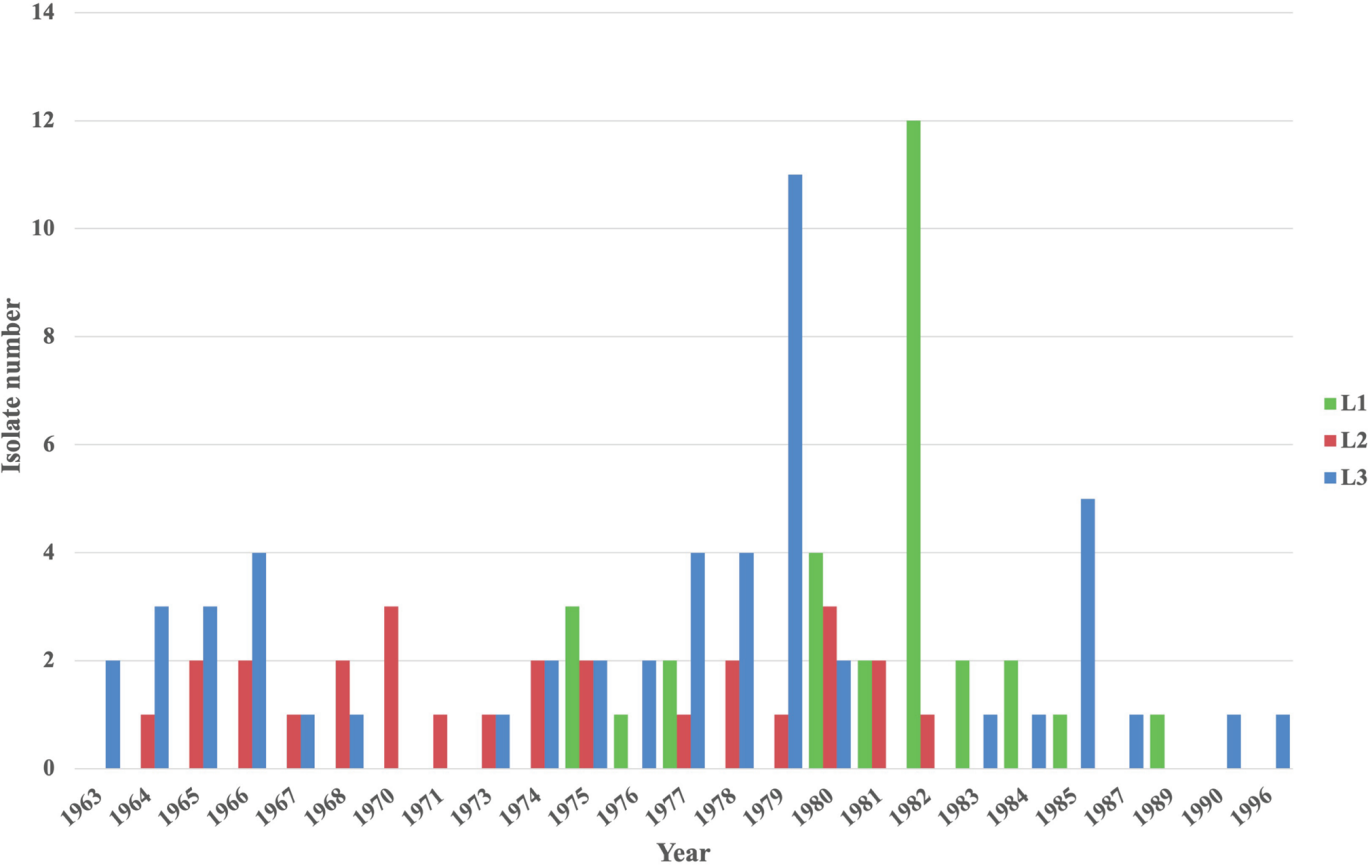
Distribution of three lineages of Zhejiang non-pandemic O1 *V. cholerae* in different years. The *y*-axis shows the isolate numbers, while the *x*-axis shows the year of isolation. The color bars are the isolate numbers in three lineages in each year as shown in the legend.

When the temporal distribution of the isolates from Zhejiang was analyzed by lineage, L1 isolates were found between 1975 and 1989, while L2 isolates were found from 1964 to 1982. No isolates of L2 were found after 1982. L3 was the largest lineage and was isolated from 1963 to 1996 (Fig. 4).

### MGT typing of Zhejiang isolates

Many isolates as shown in the phylogenetic tree were closely related (Fig. 3). To better identify closely related isolates by genotype and to determine whether any epidemiological links, all 109 genomes from Zhejiang were typed by MGT (34). STs were assigned from MGT1 to MGT7, where MGT1 is the 7-gene MLST (lowest resolution), and MGT7 is the species core genome MLST (highest resolution; Table S2). MGT6 and MGT7 level STs are shown in Fig. 3. The majority of the STs at MGT7 were singletons with only four pairs of STs containing two isolates, suggesting few isolates were epidemiologically linked. At the MGT6 level, 21 STs contained two to five isolates (Fig. 3; Fig. S3). MGT6 ST4043 had five isolates from three cities isolated in 1982, while other MGT6 STs with more than one isolate including ST4024, ST4326, ST4036, and ST4056 were all found in two different cities but with a gap of 2, 5, 7, and 16 years, respectively.

### Pan-genome analysis and lineage-specific genes

The pan-genome of the 179 genomes in this study contained 39,153 genes including 2,275 core genes as estimated using Roary (Fig. S4). We determined whether any genes were associated with the lineages using Scoary. Two genes (*ydhC_1* and *nimR_2*), annotated as encoding inner membrane transport protein YdhC and HTH-type transcriptional regulator NimR (also known as YeaM), were predicted to be present in all the three lineages and the pandemic clones except one genome of ST75, as well as two isolates outside of the lineages from Thailand. IS*Vch1* belonging to the IS*481* family transposases was found in three lineages and pandemic clones, as well as one each outlier genome from Thailand and the USA. One IS*Vha3* and nine hypothetical protein-encoding genes were present in all L1 genomes and one genome in L3. The IS*Vha3* transposase was closer to that from *Vibrio campbellii* (GenBank accession no. WP_086028432). In L2, six genes were lineage-specific with high sensitivity (100%) and specificity (>99.3%) but were all encoding hypothetical proteins. One gene, *pilA*, was found in all L3 isolates and pandemic and related clones except the Australian clone.

### *Vibrio* seventh pandemic islands (VSP-1 and VSP-2)

We analyzed the isolates of the three lineages for carriage of known pandemic-related virulence genes and genomic islands. Three isolates in L3 carried an intact VSP-1. The VSP-1 sequences from two isolates (VC1013 and VC445) were identical to the seventh pandemic reference strain, while the third (VC992) was different from the seventh pandemic as it lacked two fragments in the two neighboring VSP-1 genes (*VC0179* and *VC0180*).

The presence of the 23 VSP-2 genes (*VC0490-VC0516*) was variable across the three lineages. In L1 and L2, *VC0501* was the only gene present in most isolates. *VC0501* was present in all isolates in L2, 25.8% of L2 isolates (8/31), and only 6.7% of L3 isolates (4/59). *VC0499* was found in most L3 isolates (77.9%, 46/59). Various combinations of genes *VC0494*, *VC0496*, *VC0497*, *VC0498*, *VC0499*, *VC0504*, *VC0505*, *VC0506*, *VC0508*, *VC0509,* and *VC0510* were present in 48 of the 59 (81.3%) L3 isolates (Table S3).

### *Vibrio* pathogenic island (VPI), CTXφ, and RTX toxin genes

All genes encoding TCP pilus on the VPI (6) were present in the VPI-positive isolates. The VPI was found to be present in two of the 27 (7.4%) L2 isolates and all 59 L3 isolates, while no L1 isolates were VPI-positive (Fig. 3). We found that the sequence variation among the VPI genes was high with some genes being very divergent. To better classify the VPIs, we used protein sequences rather than nucleotide sequences to compare them, and thus we refer to the different VPI types as protein types (PTs). The VPIs from the 68 VPI-positive isolates were divided into 12 PTs (Fig. 3). There was one PT in L2 and three PTs in L3. Among these PTs, PT8 was the major type (77.97%, 46/59) in Zhejiang isolates. PT10 and PT8 were found in a cluster of 48 isolates in L3.

Two isolates in L3 carried the entire CTXφ including the *ctxAB* genes. The *ctxB* genes in these two isolates were all typed as *ctxB3*—the typical *ctxB* type of the 1961 to 1990 isolates of the seventh pandemic clone (34). Since the two isolates were located on the tree separately, clearly they acquired the CTXφ independently. Furthermore, the *ctxAB, zot*, and *ace* genes were identical to those of the seventh pandemic strain N16961.

All isolates in the three lineages contained the four genes of multifunctional autoprocessing repeat-in-toxin (MARTX) toxin gene cluster (*rtxA, rtxB, rtxC,* and *rtxD*).

### Type VI secretion system (T6SS) and type III secretion system (T3SS) genes

All isolates from this study carried 19 genes on the T6SS gene cluster including the two genes encoding T6SS effectors VgrG-2 and VgrG-3. All the T6SS gene clusters in these isolates contained the G→T T6SS-on switch as reported previously (35), which is located in the intergenic region between the two T6SS genes, *VCA0106* and *vipA* (Fig. S5), suggesting that the T6SS carried by these isolates is active.

All 31 isolates in L1, one isolate in L3, and 14 isolates outside the three lineages also carried a T3SS. The T3SS gene cluster was closely related to the T3SS2 from the *Vibrio parahaemolyticus*, but the effector genes (*vopCLP*) were absent (Fig. S6). Note that *V. parahaemolyticus* carries two T3SS gene clusters with T3SS2 referring to the one located on chromosome 2 (35). All the T3SS genes [structural genes (*vscC2J2Q2R2S2T2U2, vcr*D2), ATPase gene *vscN2*, and translocon gene *vopB2D2*) were identified in all isolates of L1 and one isolate of L3.

### Antimicrobial resistance (AMR) genes and plasmids

We screened all isolates for all AMR genes in the ResFinder database using ABRicate. There were 10 AMR genes present in the Zhejiang isolates [*aph (3”)-la_1*, *blaCARB-7_1*, *blaCARB-9_1*, *catB9_1*, *tet (35)_1*, *tet(c)_2*, *aph (3”)-lb_1*, *aph(3”)-lb_2*, *str_1*, *qac(D)_1*], in which four genes were present in only one isolate. The *bla_CARB-7_* gene was identified in 96.3% of L2 isolates (26/27), but only in one L1 isolate and three L3 isolates. The *catB9* gene was present in 91.5% (54/59) of the L3 isolates and 44.4% (12/27) of the L2 isolates (Table S4).

A total of four isolates in the three lineages were found to be positive for plasmid genes using PlasmidFinder. An L2 isolate from Zhejiang carried a *Col(pHAD28)_1* plasmid, while three L3 isolates from Japan carried an *IncC_1* plasmid (Table S4).

## DISCUSSION

### Epidemiology of three non-pandemic O1 lineages in Zhejiang

We previously examined non-pandemic O1 isolates from 2005 to 2014 in Zhejiang and found 33 O1 STs causing sporadic cholera infections with the predominance of ST75 (15). A recent study also found that ST75 was causing cholera across China (18) although that study did not report the isolates as ST75 and only referred to them as non-toxigenic O1. In this study, we analyzed historical non-pandemic O1 isolates from 1963 to 1996 by genome sequencing. *In silico* MLST divided the isolates into 12 STs with five predominant STs (ST173, ST172, ST174, ST164, and ST167). Phylogenomic analysis divided the isolates into three lineages (L1–L3). Each of the lineages had distinctive features. In particular, only two isolates in L3 carried the full complement of the key virulence factors of pandemic clones, VPI and CTX, to cause cholera. L1, comprised of predominantly ST167, carried a T3SS, and most isolates (87%, 27/31) were from human infections; L2, mainly consisted of two STs (ST164 and ST174), had no unique virulence factors, and most L2 isolates (74%, 20/27) were obtained from the environment; and L3, predominated by ST173, carried the VPI that encodes the TCP pilus, and the majority of these isolates were from human infections (66.7%, 36/54). The three lineages have different propensities to cause disease in humans.

Although there are no clinical records, the clinical symptoms caused by these non-pandemic O1 isolates may likely have differed from the typical cholera symptoms with acute watery diarrhea since most of the isolates lacked the cholera toxin genes as a key virulence factor. It is now clear that our non-pandemic O1 isolates were not differentiated from the seventh pandemic clone at the time of isolation as they shared similar biotype properties and thus were not treated differently. Some of these isolates were obtained during periods of cholera upsurges in China. During the 1980s, there was a cholera epidemic reported as “paracholera” in China (36). However, the “paracholera” epidemic was later known to be an upsurge of the seventh pandemic in China (37), as paracholera in China was generally referred to as cholera caused by El Tor strains, based on the old terminology (38).

Further typing using MGT also showed that 16 sets of non-pandemic O1 isolates shared STs at the MGT6 level, while at the MGT7 level, only four pairs shared the same STs. Identical isolates at MGT7 level (species core genome MLST) suggest that these isolates were likely to be epidemiologically linked. Among the four MGT7 STs (ST5564, ST5970, ST5604, and ST5584), each had a pair of isolates collected in the same year. Except for the ST5564 pair, all other pairs came from different cities (Fig. 3). Interestingly, the two ST5564 isolates were from humans, and from the same city and the same year, suggesting they were epidemiologically linked. Both ST5970 isolates were from the environment, while ST5970 and ST5604 each had one isolate from humans and one from the environment. As the human and the environment isolates were from different cities, it is less likely they were epidemiologically linked. Since most MGT7 STs were singletons and a small number of STs at the MGT6 level contained multiple isolates from human sources, suggesting that these non-pandemic O1 STs mostly caused sporadic cases, not large outbreaks. Isolation of the same ST from the same year but from different cities suggested that these STs had spread to different cities, likely before the year they were isolated. There must be environmental factors leading to their increased isolation in the same year.

The three lineages were distributed in eight different local government cities sporadically across 30 years. By temporal distribution of the three lineages, L3 was isolated from 1963 to 1996, the entire studied period. L2 was isolated only from 1964 to 1982. L1 emerged later than L2 in 1975 and lasted longer than L2 until 1989. In our previous study of non-pandemic O1 isolates from 2005 to 2014 (15), ST173 (L3) was observed while none of the L1 and L2 STs was found, suggesting that L3 persisted in Zhejiang causing infections in recent years. L3 and L1 caused the largest number of cases in 1979 and 1982, respectively. Interestingly, these peak years corresponded to the years of an upsurge of the seventh pandemic cholera in China (37). These isolates would possibly have been mistaken as isolates of the seventh pandemic clone. The isolation of non-pandemic O1 may also be a result of increased surveillance of cholera during this period. Furthermore, it was interesting to note that by serotype, most of the Inaba isolates were isolated from the early 1980s, superseding the Ogawa serotype. It is now clear that most of the Inaba isolates belonged to L1. Therefore, the changes in serotype in the 1980s were due to lineage replacement rather than serotype switching which was different from the switching from Ogawa to Inaba observed in the seventh pandemic clone (39).

It is unknown why L1 and L2 emerged at different times and subsequently disappeared, while L3 persisted. In our recent study of O139 from 1994 to 2018 (10), we found that there were three lineages of O139 replacing one with another successively, potentially mediated by the acquisition of AMR through mutations and plasmids. For the non-pandemic O1 isolates in this study, the majority of L2 isolates carried a *bla_CARB-7_* gene with resistance to ampicillin, which may have conferred an advantage to L2. *bla_CARB-7_* was first reported in non-O1/non-O139 isolates and was located on the superintegron (40). The chloramphenicol acetyltransferase gene *catB9* was present in the majority (89.8%) of the L3 isolates and a small proportion (44.4%) of the L2 isolates. All 12 ST174 isolates in L2 were negative for *catB9*. However, *catB9* was found in all seventh pandemic isolates and O139 isolates (10) and has not been associated with phenotypic resistance (41). Therefore, *catB9* is less likely to be associated with L2 emergence or L3 persistence in Zhejiang. On the other hand, it is possible that *catB9* was acquired by the MRCA of L3 and the seventh pandemic clone as it was present in both.

Only eight publicly available genomes fell into any of the three lineages, with one from Thailand in L1 and seven from three countries/regions (one from Taiwan, one from Vietnam, two from Haiti, and three from Japan) in L3 (Fig. 3), suggesting that these three lineages were relatively rare outside China. By contrast, the U.S. Gulf Coast clone ST75 was more widely prevalent. We previously reported ST75 isolates in 2005–2014 from Zhejiang, and we referred to it as U.S. Gulf Coast-like clone ST75b since it was a sister sublineage to the U.S. Gulf Coast sub-lineage. However, no ST75 isolates were identified in this data set with isolation years up to 1996, and thus ST75b was likely to have emerged between 1997 and 2005. A study from Taiwan identified 35 ST75 isolates in 2009–2018 (16), while another study from South Africa reported seven ST75 isolates in 2018–2020 (17). All of these isolates belonged to the ST75b sublineage. The three non-pandemic lineages identified in this study appear to have been replaced by ST75 in Zhejiang province in recent years.

The reservoir of these non-pandemic O1 clones must be the local environment as isolates for each lineage were obtained in the environment over multiple years from different cities. This situation is in contrast to pandemic O1 and O139 clones. A recent study found that the seventh pandemic cholera upsurges in Africa were associated with repeated importations rather than acquisitions from the local environmental reservoir (42). We previously also showed that the O139 outbreaks in Zhejiang were due to direct importations from overseas or spread from other parts of China rather than a local environmental origin (10). Interestingly, a sampling over 2 years (2015–2016) of river waters in two cities in Zhejiang only found non-O1/non-O139 isolates and uncovered no O1 isolates (5), suggesting that these non-pandemic O1 clones had gone extinct or were surviving in the environment with low frequency.

### Key virulence factors and pathogenicity of the three lineages

The VPI was present in all L3 and two L2 isolates in this study. The VPI was present in all epidemic and pandemic *V. cholerae* strains and sporadically in non-O1/non-O139 environmental isolates (43, 44). Since the VPI encodes the TCP as a key colonization factor and the receptor for the CTXφ (45), it is widely accepted that by acquiring the VPI and the CTXφ, an environmental strain can become pathogenic (46). We found that 88.5% of the VPI-positive isolates in L3 were of clinical origin, which is perhaps not surprising since the VPI encodes both the TCP and the ACF and must be a key pathogenicity determinant for L3.

VSP-1 and VSP-2 were markers associated with the seventh pandemic clone although their roles were unclear (47). VSP-1 was found only in the seventh pandemic *V. cholerae* isolates, but VSP-2 was also sporadically present in isolates of other *Vibrio* species (43). We found that the VSP-1 and VSP-2 gene clusters were present in a small number of isolates in this study. Two isolates in L3 carried the entire VSP-1 and nearly the entire VSP-2 with one isolate missing one VSP-2 gene and the other missing two VSP-2 genes. Our study adds to previous findings that VSP-1 and VSP-2 were present sporadically in other *V. cholerae* isolates (48) and not uniquely associated with the pandemic capability of the seventh pandemic clone.

A T3SS gene cluster was found in all L1 and one L3 isolate. Since 86.7% of the isolates in L1 were clinical O1 isolates, the T3SS must have been the key virulence factor conferring pathogenicity to L1 isolates. T3SS was found in non-O1/non-O139 *V. cholerae* causing sporadic cholera in our previous study (4) but was not found in the environmental non-O1/non-O139 *V. cholerae* (5). T3SS has also been reported in a non-O1/non-O139 *V. cholerae* strain that caused an outbreak (49). The proportion of clinical non-O1/non-O139 *V. cholerae* in India that contained T3SS was 31.5% (50). The T3SS gene cluster found in this study was closely related to the T3SS2 in *V. parahaemolyticus*, suggesting that the T3SS in our isolates originated from *V. parahaemolyticus* or acquired from a common source. However, the *V. cholerae* T3SS from L1 and L3 lacked the effector genes *vopCLP* that are present in *V. parahaemolyticus*. The T3SS gene cluster was co-located with IS*Vha3* in all L1 and the only T3SS-positive L3 isolate, suggesting that this IS element was involved in the acquisition of the T3SS gene cluster.

The T6SS gene cluster was found in all isolates in this study. For the variants of the effector gene *vgrG*, apart from *vgrG-3* that is located in the main gene cluster, four other variants (*vgrG-1*, *vgrG-2*, *vgrG-4*, and *vgrG-5*) are known to be present in various strains (51). Our isolates also carried an additional gene cluster that encodes the Vgr*G-2* effector but not the other *vgrG* variants. T6SS was reported to be active in environmental *V. cholerae* isolates but inactive in pandemic isolates (52). The SNP (G→T) located upstream of *vipA* reported as a switcher of active and inactive state (35) was found in our isolates suggesting that the T6SS is active in these isolates.

### Relationship of non-pandemic O1 strains to pandemic clones

The elucidation of the relationships of O1 lineages in this study also gave us a better view of the evolutionary origin of the pandemic clones. The sixth and the current seventh pandemic clones were represented by ST73 and ST69/ST515, respectively (12, 14). In addition to the pandemic clones, there are other non-pandemic strains closely related to the seventh pandemic clone including the Australian clone, the U.S. Gulf Coast clone, and the pre-seventh pandemic clone belonging to ST70, ST75, and ST71, respectively (12). These O1 clones shared the MRCA with the seventh pandemic clone (53) and the sixth pandemic clone and formed a separate clade from the other non-pandemic O1 strains in this study (Fig. 3). Thus, the two pandemic clones shared the MRCA and were more closely related than previously thought (54). Interestingly, the three newly found non-pandemic lineages in this study were grouped together with the pandemic clones without any major non-O1 lineages in between them (Fig. S1). Clearly, all these lineages were related. L1 shared the MRCA with both L2 and L3, while L3 shared the MRCA with the pandemic clones (Fig. 3).

Since all these lineages were O1, it seems plausible that the ancestor of these lineages obtained the O1 antigen gene cluster and then the MRCA of L3 and pandemic and related clones acquired VPI as all their decedents carried VPI. Although the VPI diversity was quite high with different PTs seen among the isolates, the VPI was likely to have been acquired as a single event that allowed the pandemic clones to colonize human hosts. The MRCA of the pandemic and related clones subsequently acquired the CTX, to gain the capability of causing cholera and pandemics.

The acquisition of other genes by the ancestor of these non-pandemic lineages may have also contributed to their success as human pathogens. One gene *pilA*, encoding a component of a chitin-regulated pilus (ChiRP), was found uniquely in L3 and pandemic-related clones including sixth, seventh, and U.S. Gulf Coast clones. ChiRP is a type IV pilus and found to contribute to the colonization process of *V. cholerae* (54).

Two genes, *ydhC_1* and *nimR_2*, encoding the transporter factor (TF) YdhC and regulator NimR, respectively, were found to be present in all three non-pandemic lineages and the pandemic and closely related clones. YdhC was hypothesized as a purine-related efflux pump that responds inversely to purine biosynthetic gene expression (55) and was further inferred to contribute to adenosine efflux (56). NimR was identified as a transporter regulator of 2-nitroimidazole in *E. coli* (57). It would be interesting to determine the roles of these genes in facilitating the pathogenicity of *V. cholerae* pandemic clones and the non-pandemic lineages.

### O1 diversity and definition of epidemic clones

There is a well-established division of O1/O139 and non-O1/non-O139 *V. cholerae* with the former capable of causing epidemic- and pandemic-level cholera and the latter causing only sporadic cholera (58). Our study showed that there is a clear demarcation of the pandemic and related O1 clones and non-pandemic O1 clones phylogenetically. Among the 65 publicly available non-pandemic O1 genomes, there were an additional 57 falling outside of the three lineages we identified in this study, clearly showing that more diversity of non-pandemic O1 strains exists. Differentiating them is useful for epidemiological purposes and the assessment of potential public health risks of existing or emerging non-pandemic clones. The separation of pandemic clones from non-pandemic clones by O antigen only is clearly inadequate. MLST can unambiguously distinguish pandemic and closely related O1 clones and unrelated O1 clones from each other, with the current seventh pandemic clone being marked by two STs, ST69 and ST515. However, the epidemic O139 clone was derived from the seventh pandemic clone, and they share the same ST, ST69, which can be further differentiated using MGT as described previously (34). Their separation would require both MLST and O antigen typing. Similarly, the sixth pandemic clone and its O37 derivative shared the same ST although both seem to be extinct (12). The frequent transfer of the O antigen gene cluster and other virulence genes and the high level of recombination in *V. cholerae* can lead to the rapid emergence of new clones and complicate public health surveillance of *V. cholerae*. Genome sequencing ultimately offers the best resolution but may not be readily available or economical as a surveillance tool in developing countries, wherein the burden of cholera disease is higher. Lineage-specific genes identified in this study may help differentiate and track these lineages using PCR upon further development.

### Conclusion

Non-pandemic *V. cholerae* O1 isolates in Zhejiang were divided into three lineages. Each lineage has a distinctive propensity to cause disease in humans. L3 persisted in Zhejiang from the 1960s until recently. L1 and L2 emerged at different times but were not found after the 1990s. The three lineages were replaced by a U.S. Gulf Coast-like clone ST75b in Zhejiang (15). Based on the absence of CTX in most isolates from the three lineages, they were unlikely to cause typical cholera. VPI and T3SS in L3 and L1, respectively, were the key virulence factors found that may enable them to cause disease in humans. The VSP islands were found to be present in our non-pandemic O1 isolates, suggesting that the VSPs are not uniquely associated with the seventh pandemic clone. One of the three non-pandemic lineages (L3) shared, the MRCA with the pandemic clones but diverged when pandemic clones acquired CTX as a key event to become toxigenic to humans and cause typical cholera disease. A combined O antigen typing and MLST strain typing would be required to differentiate the pandemic from non-pandemic clones. This study provided a better understanding of the evolution of O1 non-pandemic clones and their relationship to pandemic O1 clones.

## Data Availability

All genome sequences in this study have been submitted as raw reads under BioProject accession number PRJNA646107 in the NCBI SRA database.

## SUPPLEMENTAL MATERIAL

Fig. S1 to S6 spectrum.02615-23-s0001.pdf Supplemental figures.

Table S1 to S4 spectrum.02615-23-s0002.xlsx Supplemental tables.

ASM does not own the copyrights to Supplemental Material that may be linked to, or accessed through, an article. The authors have granted ASM a non-exclusive, world-wide license to publish the Supplemental Material files. Please contact the corresponding author directly for reuse.
