# Supplementary material for: Emergence and genomic insights of non-pandemic O1 Vibrio cholerae in Zhejiang, China
Source: Microbiol Spectr. 2023 Oct 11;11(6):e02615-23. doi: 10.1128/spectrum.02615-23 (PMC10871787; doi:10.1128/spectrum.02615-23)
Supplement: Fig. S1 to S6 — Supplemental figures. [file spectrum.02615-23-s0001.pdf]

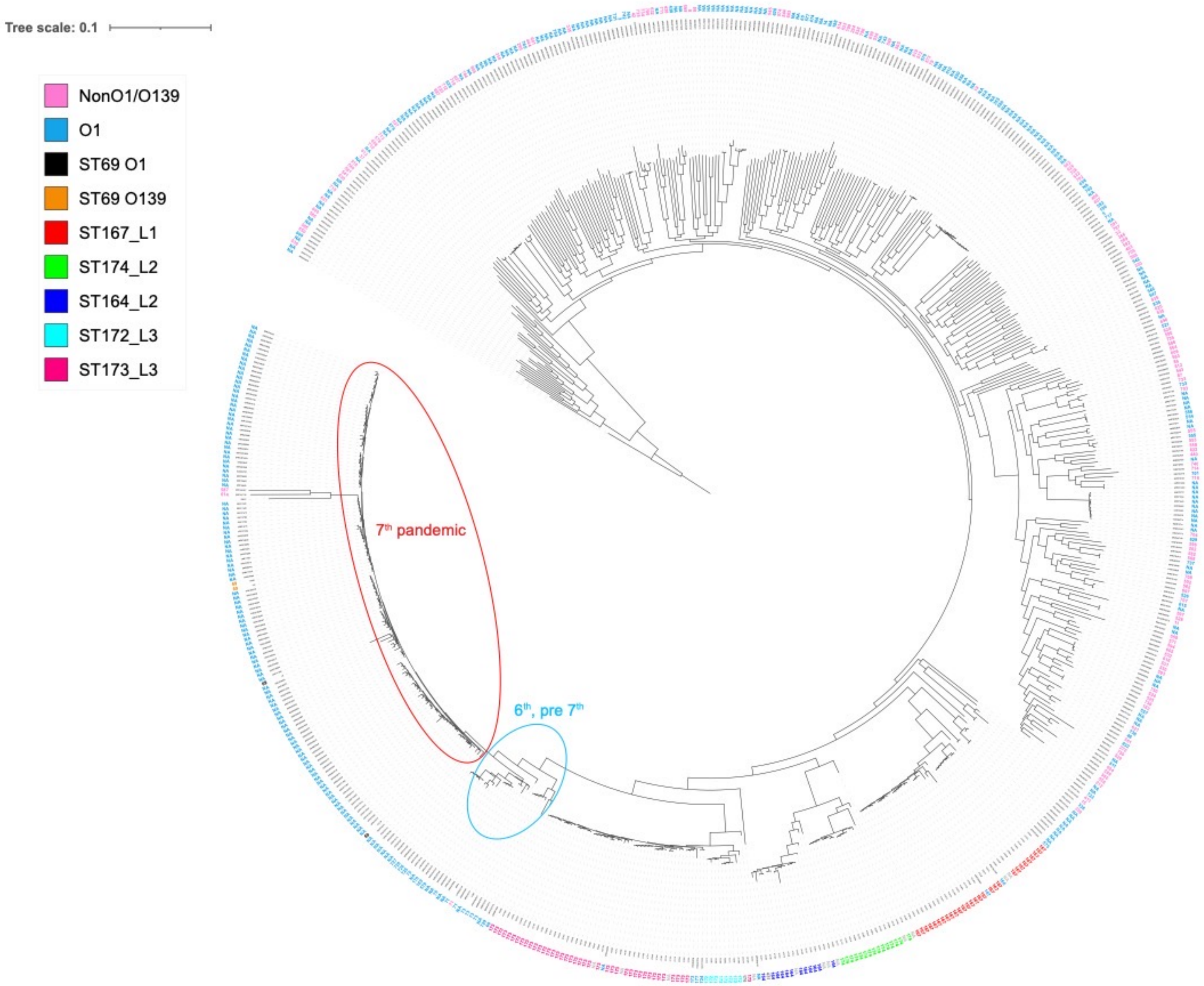

Fig. S1. Phylogenetic tree of Non 7th pandemic *V. cholerae* O1 genomes compared with other *V. cholerae* serogroups
Different STs and serogroups are labeled in different colors as showed in the legend.

A

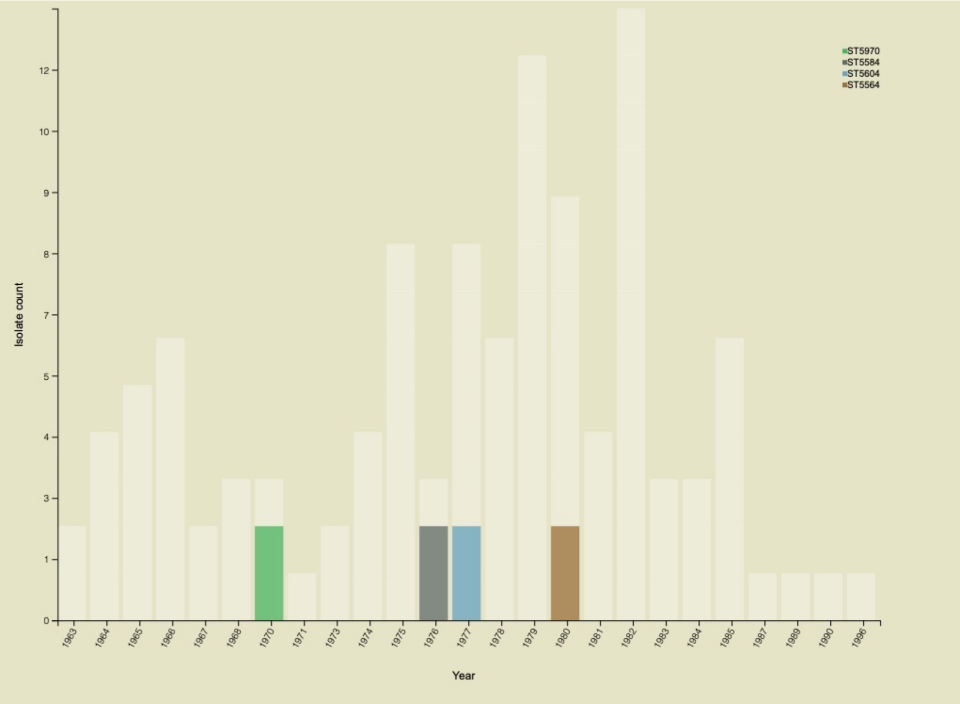

B

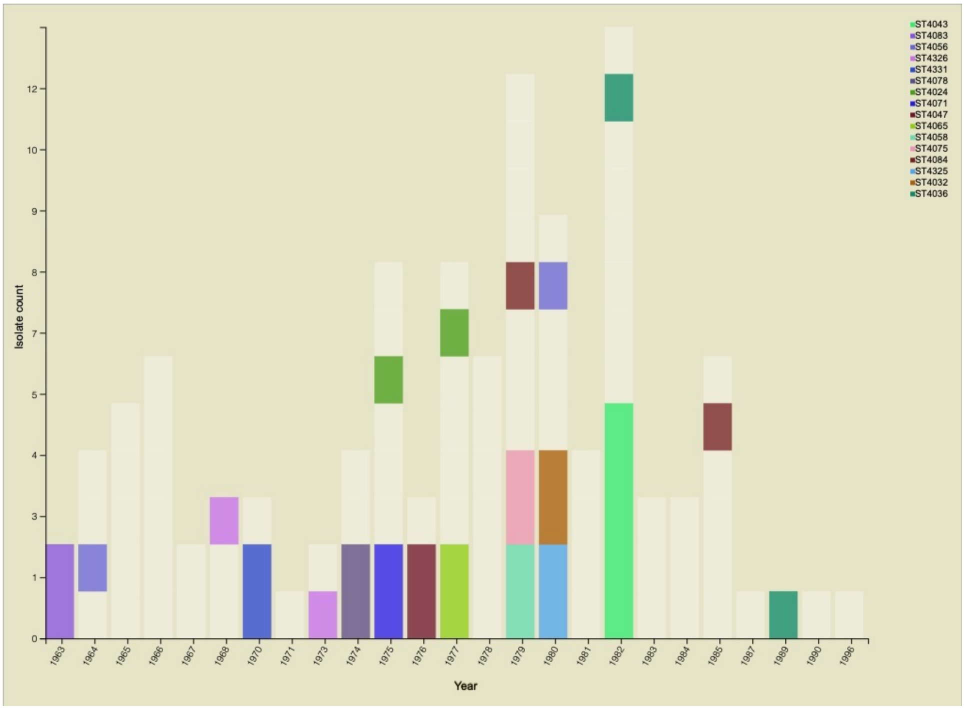

Fig. S3. Distribution of ZJ isolates with MGT STs.

A. Four non-singleton MGT7 STs were assigned. White indicates singletons.
B. Distribution of 16 non-singleton MGT6 STs of ZJ isolates.

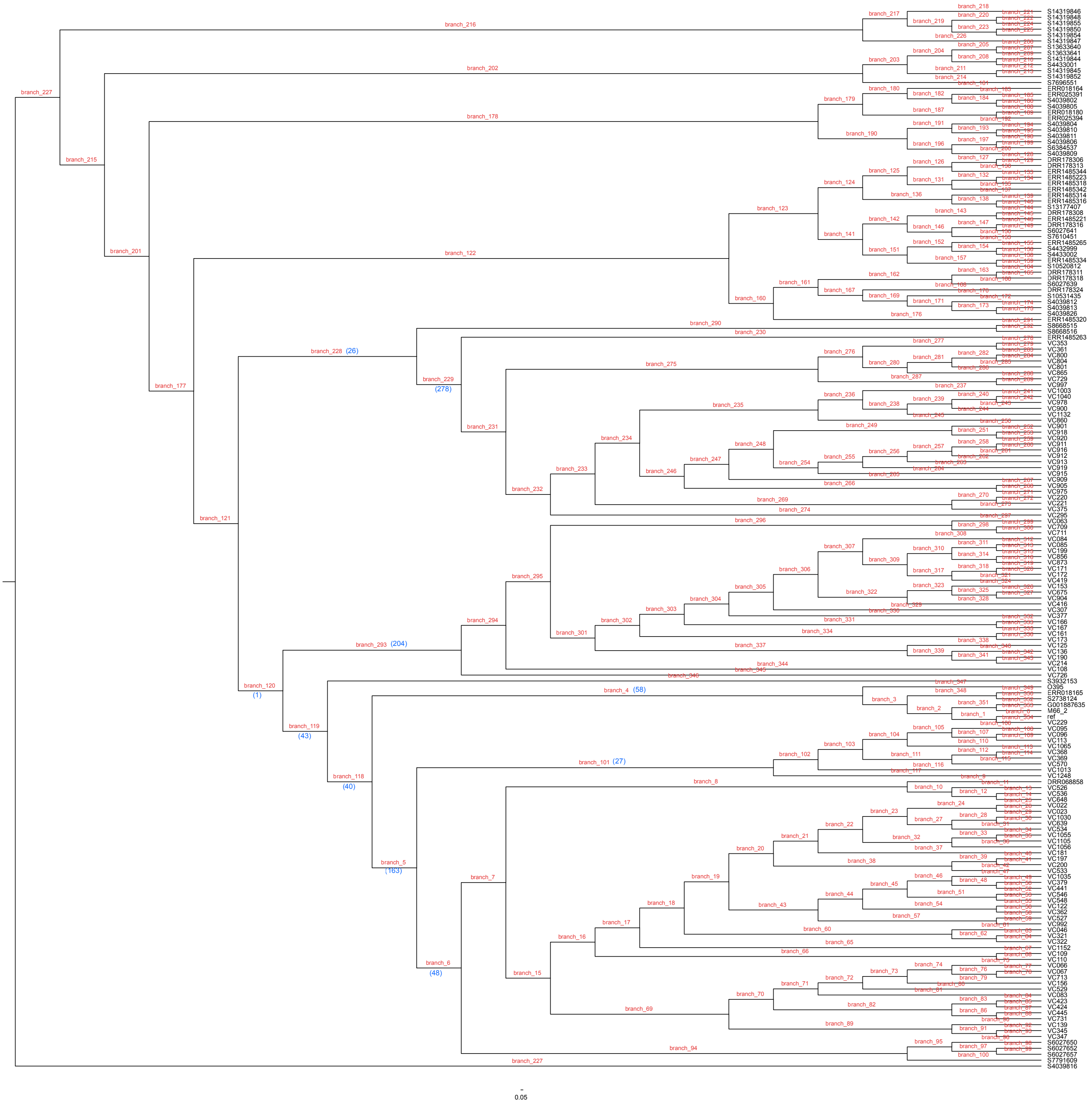

Fig. S2. Branch ID and supporting SNPs one major branches. Key branches were marked with a branch number followed by brackets of the blue number of SNPs that supported the branch.

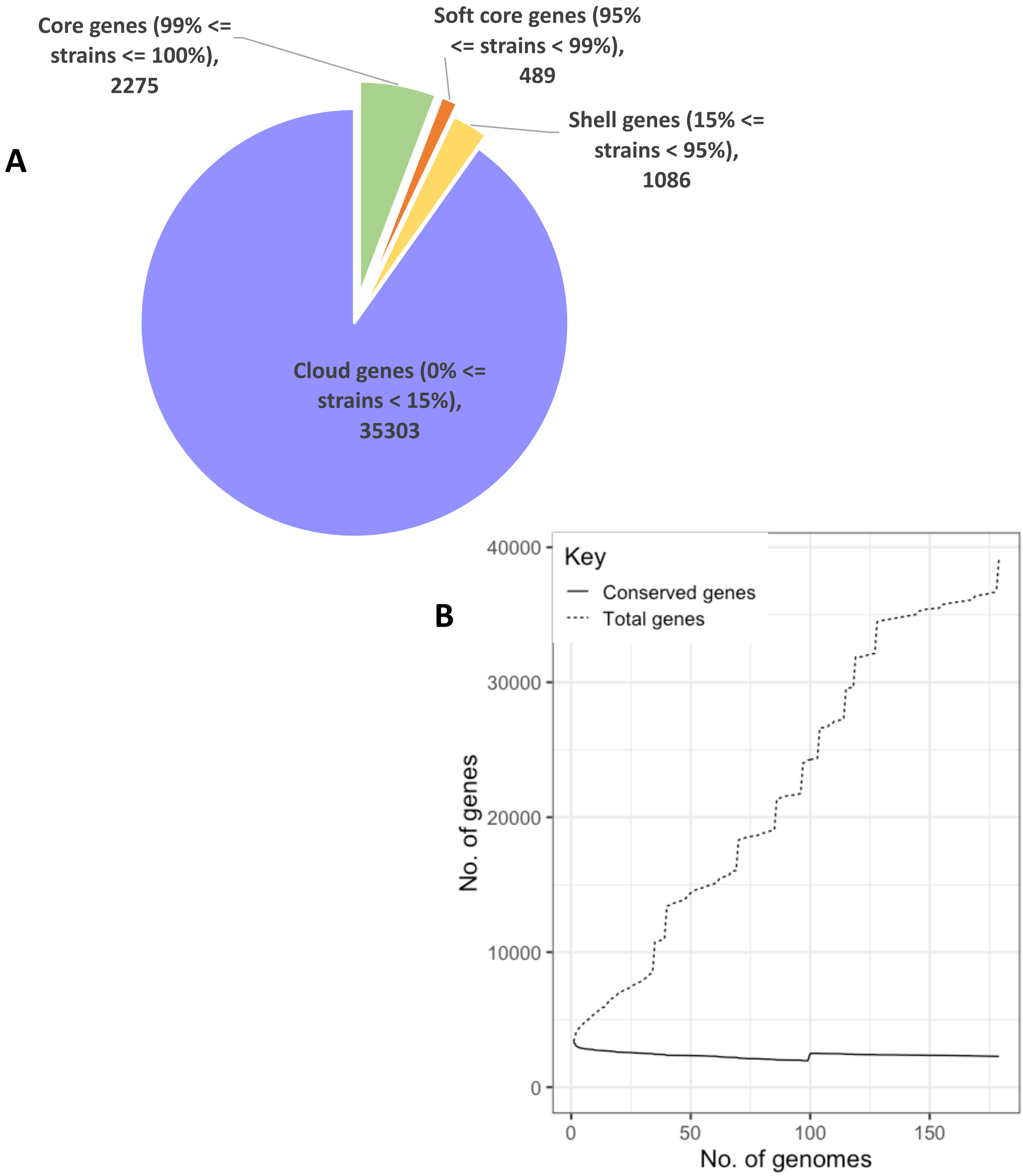

Fig. S4. Non 7th pandemic O1 *V. cholerae* pan-genome.
(A) Pan and core genes estimated by Roary. (B) The number of core genes (solid line) and pan genes (dash line) is plotted as a function of the number of genomes sequentially added.

VC185_concat	TTCCCTGTTAAGTATTATTTAAGTATGGTTATTGGATATATTTTTGGGTAAAAGATCACGCTTCGG
VC190_concat	TTCCCTGTTAAGTATTATTTAAGTATGGTTATTGGATATATTTTTGGGTAAAAGATCACGCTTCGG
VC197_concat	TTCCCTGTTAAGTATTATTTAAGTATGGTTATTGGATATATTTTTGGGTAAAAGATCACGCTTCGG
VC199_concat	TTCCCTGTTAAGTATTATTTAAGTATGGTTATTGGATATATTTTTGGGTAAAAGATCACGCTTCGG
VC200_concat	TTCCCTGTTAAGTATTATTTAAGTATGGTTATTGGATATATTTTTGGGTAAAAGATCACGCTTCGG
VC210_concat	TTCCCTGTTAAGTATTATTTAAGTATGGTTATTGGATATATTTTTGGGTAAAAGATCACGCTTCGG
VC214_concat	TTCCCTGTTAAGTATTATTTAAGTATGGTTATTGGATATATTTTTGGGTAAAAGATCACGCTTCGG
VC220_concat	TTCCCTGTTAAGTATTATTTAAGTATGGTTATTGGATATATTTTTGGGTAAAAGATCACGCTTCGG
VC221_concat	TTCCCTGTTAAGTATTATTTAAGTATGGTTATTGGATATATTTTTGGGTAAAAGATCACGCTTCGG
VC229_concat	TTCCCTGTTAAGTATTATTTAAGTATGGTTATTGGATATATTTTGGGGTAAAAGATCACGCTTCGG
VC295_concat	TTCCCTGTTAAGTATTATTTAAGTATGGTTATTGGATATATTTTTGGGTAAAAGATCACGCTTCGG
VC307_concat	TTCCCTGTTAAGTATTATTTAAGTATGGTTATTGGATATATTTTTGGGTAAAAGATCACGCTTCGG
VC321_concat	TTCCCTGTTAAGTATTATTTAAGTATGGTTATTGGATATATTTTTGGGTAAAAGATCACGCTTCGG
VC322_concat	TTCCCTGTTAAGTATTATTTAAGTATGGTTATTGGATATATTTTTGGGTAAAAGATCACGCTTCGG
VC345_concat	TTCCCTGTTAAGTATTATTTAAGTATGGTTATTGGATATATTTTTGGGTAAAAGATCACGCTTCGG
VC347_concat	TTCCCTGTTAAGTATTATTTAAGTATGGTTATTGGATATATTTTTGGGTAAAAGATCACGCTTCGG
VC353_concat	TTCCCTGTTAAGTATTATTTAAGTATGGTTATTGGATATATTTTTGGGTAAAAGATCACGCTTCGG
VC361_concat	TTCCCTGTTAAGTATTATTTAAGTATGGTTATTGGATATATTTTTGGGTAAAAGATCACGCTTCGG
VC362_concat	TTCCCTGTTAAGTATTATTTAAGTATGGTTATTGGATATATTTTTGGGTAAAAGATCACGCTTCGG
VC368_concat	TTCCCTGTTAAGTATTATTTAAGTATGGTTATTGGATATATTTTTGGGTAAAAGATCACGCTTCGG

Fig. S5. The intergenic sequence between VCA0106 and vipA from the isolates in this study.
SNP45 was ‘G’ in VC229 which was an 7th pandemic isolate while other SNPs on position 45 were all identified as ‘T’ which was a T6SS-on switch.

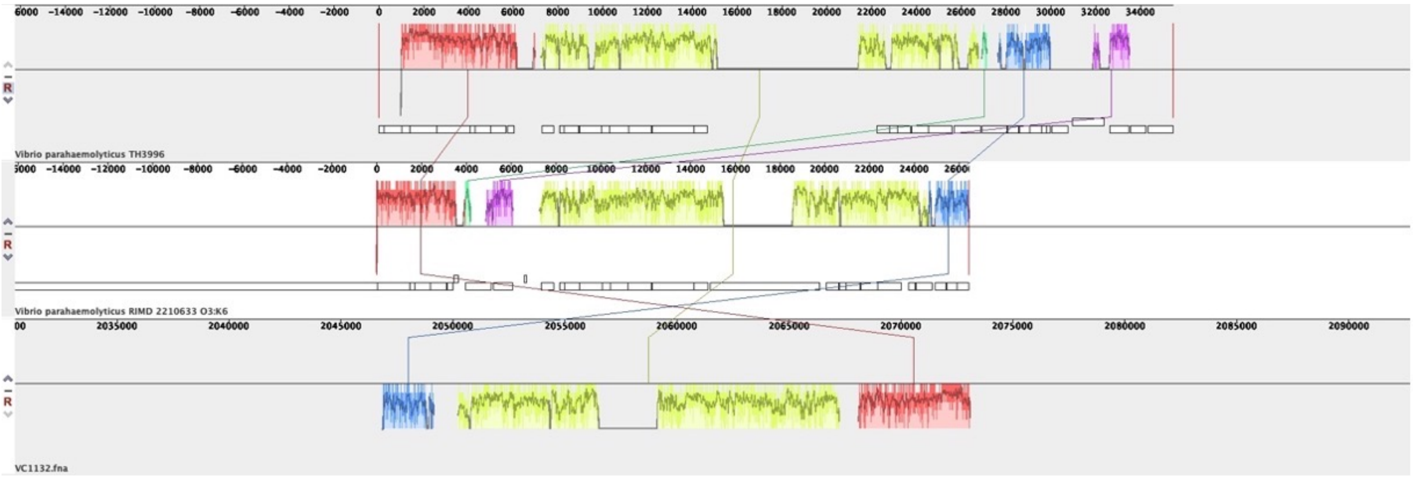

Fig. S6. Comparison of T3SS sequences in *V. parahaemolyticus* TH3996, *V. parahaemolyticus* RIMD2210633 and *V. cholerae* (VC1003) in L2.

The genes in purple are the effector vopP and vopL which are next to vscJ2 in TH3996 and between vscR2 and Q2 in RIMD2210633 but lack in VC1003.
